# Giant cell tumor of the cervical spine: A case report

**DOI:** 10.1016/j.ijscr.2024.110676

**Published:** 2024-11-29

**Authors:** Omar Abdulaziz Alolayan, Abdulrahman Jalwi Korkoman, Abdullah Adel Alnasser

**Affiliations:** a Orthopedic Surgery Department at Prince Sultan Military Medical City, Riyadh, Saudi Arabia; b Orthopedic Surgery Department at the University of Bisha, Bisha, Saudi Arabia

**Keywords:** Giant cell tumor, Cervical spine, Denosumab, Corpectomy, Case report

## Abstract

**Introduction:**

A Giant Cell Tumor (GCT) of the bone is a locally osteolytic tumor made up of mononuclear ovoid stromal cells and multinucleated giant cells. It commonly affects long bones like the distal femur and proximal tibia, but can also develop in the cervical spine during the third and fourth decades of life.

**Presentation of case:**

A 20-year-old female presented to the clinic with a complaint of neck pain persisting for one month. Clinical examination revealed bilateral radiculopathy in the upper limb, which was also associated with pain in the left shoulder and elbow. Imaging revealed a destructive lesion in the 7th cervical vertebra (C7). A biopsy was done which indicated a giant cell tumor (GCT). A C7 corpectomy was performed, which included the removal of the C7 vertebral body and application of a cage. At the two-year follow-up, the patient remained asymptomatic, and radiographs showed no signs of recurrence.

**Discussion:**

A Giant Cell Tumor (GCT) occurring above the sacrum, especially in the cervical spine, is extremely rare. Symptoms of GCT are often nonspecific and can include neck pain, numbness, and weakness in the upper limbs, which are sometimes mistaken for myalgia or disc disease. The main treatment goal for GCT is “en bloc” resection while preserving the joint, though this can be challenging, particularly in spinal cases.

**Conclusion:**

It is crucial to acknowledge that GCT can appear in uncommon sites and age groups. Surgeons must have a high level of awareness and perform an adequate preoperative workup, before definitive treatment. The patient should undergo regular follow-ups to detect any recurrence or metastasis at an early stage.

## Highlights

• Giant Cell Tumor (GCT) is a locally osteolytic bone tumor with ovoid stromal cells and multinucleated giant cells.
• Giant Cell Tumor (GCT) affects long bones (distal femur, proximal tibia) and sometimes the cervical spine in ages 30–40.
• Managing GCT is challenging due to the absence of reliable clinical or radiological predictors for recurrence or metastasis.
• The primary goal in GCT management is wide resection with adjuvant therapy to reduce the high recurrence rate.

## 1 Introduction

Giant Cell Tumor (GCT) of the bone is characterized as a locally osteolytic tumor composed of mononuclear ovoid stromal cells and multinucleated giant cells. GCT typically occurs in long bones such as the distal femur, proximal tibia, and distal radius [1], but it can also manifest in the cervical spine during the third and fourth decades of life [2]. Most patients experienced pain or neurological deficits at the tumor site, with symptoms often present for many months before diagnosis. The most common symptoms were pain and neurological deficits in the affected area, observed in nearly all patients [12]. Managing GCT is challenging due to the lack of definitive clinical or radiological indicators for predicting recurrence or metastasis [3]. The primary goal in GCT management involves wide tumor resection combined with adjuvant treatment to mitigate the high recurrence rate, requiring orthopedic surgeons to balance recurrence reduction with maintaining maximum function [3]. Radiotherapy has also been shown to significantly reduce recurrence rates. This suggests that it plays a crucial role not only in cases where surgery is not an option, but also as an effective complement to surgery in decreasing the risk of recurrence [14]. Due to the relatively complex anatomy of the cervical spine, particularly in the upper region, the intricate blood supply and neural structures present significant challenges for surgeons, making complete tumor removal very difficult [12]. Denosumab, an alternative to wide resection, remains controversial; a study reported tumor regrowth in 40 % of patients eight months after discontinuation of Denosumab, with side effects including hypophosphatemia, osteonecrosis, and anemia [3]. Resecting GCT in the cervical spine is particularly challenging when the vertebral artery is in close proximity, impacting tumor excision and contributing to a high recurrence rate [5]. We report the case of a 20-year-old female with a 7th cervical vertebra GCT to raise awareness among clinicians about the presence of intraosseous GCTs in rare locations and atypical age groups. This underscores the necessity of early intervention to prevent complications. The work has been reported in line with the SCARE criteria [13].

## 2 Case presentation

A 20-year-old female presented to the clinic with a complaint of neck pain persisting for one month. On clinical examination, there were no surgical scars, skin defects or muscle atrophy. There was tenderness in the lower cervical spine. Muscles power were 5/5 for bilateral upper limbs. However, she had a bilateral radiculopathy in the upper limbs, along with pain in the left shoulder and elbow and decreased sensation in the palm. Her pain was 8/10 with minimal improvement with analgesia. Additionally, she has a history of dysphagia for the past four months. There were no constitutional symptoms, and she reported no history of previous trauma. The patient was mobilizing well.

**Fig. 1:**
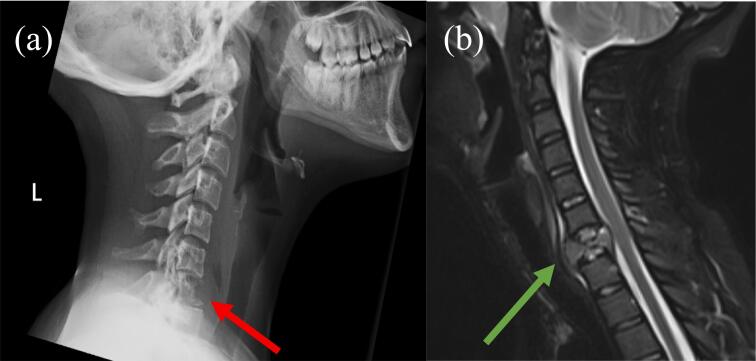
Preoperative radiographic assessment of the lesion. (a) Lateral X-ray of the cervical spine showing bone destruction at the anterior aspect of the vertebral body of C7. (b) An MRI showing a destructive lesion with vertebral plana involving C7 with associated mild cord comression.

Bone destruction at the anterior aspect of the vertebral body of the C7 on x-ray (Fig. 1a). A destructive lesion with vertebra plana involving C7 associated with enhancing prevertebral left paravertebral, and epidural soft tissue components with mild cord compression on MRI (Fig. 1b).

**Fig. 2:**
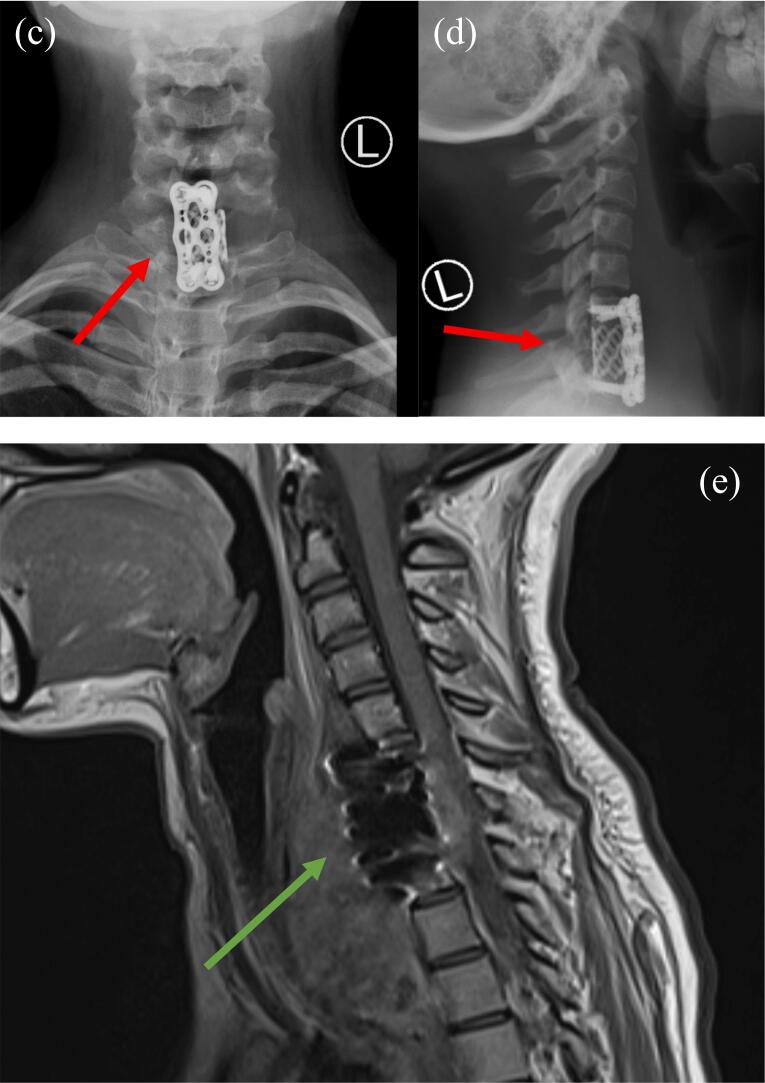
Postoperative radiographic assessment of the procedure and after the patient received radiotherapy. These images were taken after the treatment had been fully administered.

An MRI detailed the lesion's features (Fig. 1b), and subsequent evaluation, including a bone scan and CT Angio, confirmed the presence of a lytic lesion with a prevertebral soft tissue component. All biochemical investigations were normal. A biopsy was done which showed a giant cell-rich neoplasm with numerous multinucleated giant cells and epithelioid/spindle stromal cells. Mild nuclear atypia and frequent mitotic figures (∼20 mitoses per 10 HPFs) are present but are typical of a giant cell tumor (GCT) of bone. No sarcomatous changes or atypical mitosis are observed. Overall findings are consistent with a giant cell tumor of bone. A subsequent barium study revealed a posterior indentation in the lower cervical esophagus that corresponded to the known lesion. After obtaining informed consent, the patient was shifted to the operating room. A C7 corpectomy was performed, The patient was positioned supine. The superior and inferior poles of the neck were identified, and a horizontal midline incision was made. The platysma was identified and divided, followed by undermining of the platysma and deep blunt dissection. The sternocleidomastoid (SCM) and strap muscles were identified, and dissection was performed in the plane medial to the SCM and lateral to the strap muscles. A deep interval was developed lateral to the esophagus and medial to the carotid, with division of the prevertebral fascia. Dissection of the longus colli muscle off the spine was carried out, and the C7 vertebra was identified. A corpectomy was performed to remove the tumor. A cage was applied (Fig. 2c & d) with 5 cc of bone graft, and a size 28 plate with 4 × 16 mm fixed-angle locking screws was applied, An MRI was done after the procedure (Fig. 2e). The timing of the patient's surgery was carefully determined as part of a multidisciplinary approach. The decision was made in collaboration with various specialists to ensure that the timing would optimize the patient's treatment outcome.

The patient was discharged on the seventh day with a scheduled tumor board review. The tumor board's decision was to start the patient on radiotherapy. The patient successfully completed a total of 25 sessions. Follow-up imaging showed lesion regression. The patient was monitored every three months for the first year, followed by biannual check-ups. At the two-year follow-up, the patient was free of symptoms, and radiographs revealed no signs of recurrence.

## 3 Clinical discussion

The occurrence of a Giant Cell Tumor (GCT) above the sacrum, particularly in the cervical spine, is exceedingly rare, constituting approximately 0.4 % to 1.0 % of cases [6]. The signs and symptoms of GCT are often nonspecific and may include neck pain, numbness, and weakness in the upper limbs, which can be mistaken for myalgia or disc disease [5]. Histologically, GCT is characterized by multinucleated giant cells, stromal cells, and occasionally spindle cells [7]. Multinucleated giant cells arise from mononuclear neoplastic cells due to the overexpression of receptor activator of nuclear factor κB ligand (RANKL), leading to the recent use of Denosumab as a treatment option. Denosumab inhibits RANKL, thereby preventing osteoclast maturation and halting bone destruction [8]. In addition to Denosumab, a case study of a 46-year-old male with C5-C6 GCT treated with bisphosphonates demonstrated clinical and radiological improvement along with lesion regression over a three-year follow-up period [9]. However, surgery remains the primary treatment for GCT [9]. Megavoltage radiation has also proven effective, particularly in inoperable patients, as shown in a study conducted between March 1973 and March 1992 [10]. Despite a range of treatment options, the recurrence rate remains high, reaching 25 % to 45 % [5]. Radiologically, GCT appears as a lytic lesion near the articulate surface with well-defined margins, occasionally exhibiting aggressive features like soft-tissue destruction or cortical expansion [11]. The differential diagnosis includes simple aneurysmal cysts or osteosarcoma [5]. The primary goal in GCT treatment is “en bloc” resection with joint preservation; however, challenges may arise, particularly in spinal cases.

## 4 Conclusions

Giant Cell Tumor (GCT) of the cervical spine is exceedingly rare and typically presents as pain. Although it is generally observed in individuals in their fourth decade of life, it can occur in atypical age groups, as demonstrated in our case. A high level of awareness among surgeons, along with an adequate preoperative workup including biopsy is essential for identifying unusual tumors in atypical locations. GCT of the cervical spine is associated with high recurrence rates and potentially aggressive behaviour. Early detection and intervention can prevent local tumor spread and avoid more invasive procedures, such as corpectomy. Close follow-up is necessary to detect any recurrence or metastasis at an early stage.

## Consent

Written informed consent was obtained from the patient for publication of this case report and accompanying images. A copy of the written consent is available for review by the Editor-in-Chief of this journal on request.

## Declaration of generative AI and AI-assisted technologies in the writing process

Nothing to disclose.

## Ethical approval

This case reported is exempted to have an ethical approval because the case report is exempted from ethical approval once you secured a consent from the patient, as per our institution IRB.

## Funding

This case report did not receive any specific grant from funding agencies in the public, commercial, or not-for-profit sectors.

## Author contribution

All authors contribute in patient's examination, surgical intervention, secure the consent & writing this paper.

## Guarantor

Dr. Omar Abdulaziz Alolayan

## Research registration number

N/A.

## Conflict of interest statement

The authors have no conflicts of interest to declare. All co-authors have seen and agree with the contents of the manuscript and there is no financial interest to report. We certify that the submission is original work and is not under review at any other publication.
